# Bioinformatic analysis combined with immune infiltration to explore osteoarthritis biomarkers and drug prediction

**DOI:** 10.1097/MD.0000000000038430

**Published:** 2024-06-21

**Authors:** Yan Liu, Wei Jiang, Juan Huang, Li Zhong

**Affiliations:** a Gerontology Medicine Department, The Affiliated Hospital of Southwest Medical University, Southwest Medical University, Luzhou, China; b Rehabilitation Medicine Department, The Affiliated Hospital of Southwest Medical University, Southwest Medical University, Luzhou, China.

**Keywords:** biomarkers, drug prediction, immunity, inflammation, osteoarthritis

## Abstract

Along with global aging, osteoarthritis (OA) appears to have a high incidence and disability rate, which seriously affects the quality of life of patients, making age a major risk factor. However, the pathology of OA is under-researched, and there is no obvious and effective treatment. Research has demonstrated the importance of aging, inflammation, and immunology in the onset and course of OA. This study aims to anticipate therapeutic drugs based on critical genes associated with OA and to elucidate the roles of genes and possible biomarkers associated with inflammation, immunology, and cellular senescence in OA. The OA gene expression matrix was first obtained from the Gene Expression Omnibus database. Screening for OA significant differentially expressed genes by bioinformatics identification. Specific biological processes and related signaling pathways of the differential genes were enriched. Then elucidate the status of immune cell involvement in OA based on immune infiltration analysis. Finally predict therapeutic agents based on pivotal genes. A total of 198 differentially expressed genes were identified in OA, and TP53, EGFR, TGFB1, LEP, CD4, MAPK8, SCARB1, ADIPOQ, JAK2, and SERPINE1 were further identified as important hub genes. The enrichment results showed that the development of arthritis was mainly related to immune cell differentiation, amino acid metabolism and cellular senescence process. The validation of immune infiltration results indicated that NK_cells, CD4_Tcells, Macrophages, Monocytic_lineage, Dendritic_cells, Basophils, CD8+_naive_T-cells may play an important role in the immune process of OA. Key Drug Prediction of Hub Genes found that Halicin, Ruxolitinib, Tofacitinib, Clenoliximab, Baricitinib may be a key drug or component in the treatment of OA.

## 1. Introduction

Osteoarthritis (OA) is the most common musculoskeletal disease, which is an inflammatory disease that accumulates throughout the body in the bone and joints. It is estimated that more than 500 million people worldwide suffer from OA, with more women than men, and most of the patients are over 60 years old.^[1]^ Apart from age, one of the major modifiable risk factors that may be controlled is obesity. According to research, for every unit rise in body mass index, there is an approximate 8% increase in the risk of OA.^[2]^ Additional risk factors for OA include genetics, accident history, deterioration of the muscles, and participation in particular activities and vocations. Additionally, the onset of OA has been linked to several chronic illnesses, including diabetes and cardiovascular disease.^[3]^ While articular cartilage and synovium degradation or destruction has often been thought to be the cause of arthritis, a chronic and aseptic inflammatory is still the fundamental pathognomonic aspect of OA.^[4]^ In addition, antigens from injured joints cause inflammation by activating inflammatory vesicles, and elevated levels of senescent molecules and systemic and local inflammatory cytokines encourage the breakdown of cartilage. The effectiveness of this inflammatory factor inhibitor in treating OA, regardless of the presence of active synovitis, has to be further substantiated. As evidenced by the lack of significant improvement in pain, synovitis, or bone marrow lesions observed in several studies evaluating conventional anti-inflammatory drug therapies in inflammatory arthritis.^[5,6]^ Consequently, further study is required to determine the processes of arthritis and the most effective treatment options when combined with other metabolic illnesses. Furthermore, alterations in immune cell types associated with localized chronic inflammation have been observed by other studies, indicating the potential role of immunological reactivity in joint injury.

Activation of the immune system in the joint is the first step in the OA process, which also involves many cytokines and mediators. Particularly important in this process are “pathogen-associated molecular patterns” and “damage-associated molecular patterns”, which are tiny pieces of articular cartilage broken down and released into the articular interstitial space.^[7]^ This promotes the production of immune and inflammatory responses like chemokines and immune cell aggregation. This process involves both the innate and acquired roles of the immune system. The innate immune system is the host’s response to pathogen-associated molecular patterns, which are produced by interacting with pattern recognition receptors (PRRs) on a variety of immune cells, including monocytes, dendritic cells, neutrophils, and macrophages, that have gathered in the joint.^[8]^ Alternatively, a variety of small molecules or fragments (hyaluronic acid, proteoglycans, and proteins) generated by chondrocytes, synovial cell, and extracellular matrix as a result of damage and apoptosis may be recognized as damage-associated molecular patterns by pattern recognition receptors on immune cell membranes, which in turn trigger downstream inflammatory signaling pathways.^[9]^ This implies that one of the factors pertaining to the advancement of arthritis that must be examined is cell death.^[10]^ This might have something to do with the body’s aberrant general metabolic status, which causes cellular abnormalities in the joints and many fatalities.

Bioinformatics analysis is based on gene microarray or high-throughput sequencing microarray data from tissue cells, combining informatics and expression profiling techniques to mine valuable genes from tens or hundreds of thousands of genes to parse the underlying molecular mechanisms of diseases. The GEO database contains microarray or high-throughput expression profiling data shared by research institutes around the world, and it belongs to the public databases that have received ethical approvals.^[11]^ Based on bioinformatic analysis, we extracted the expression profiles of relevant genes between no-osteoarthritis (NA) and OA tissues by referencing a public OA sequencing dataset and assessed the relevant signaling pathways and immune cell infiltration in OA. In addition, we analyzed drugs that may modulate the hub genes through database prediction of drugs. Taken together, our data may provide new strategies for the diagnosis and treatment of OA.

## 2. Materials and methods

### 2.1. Microarray data sources and differential gene analysis

Bioinformatic data of OA were obtained from NCBI Gene Expression Public Database. The dataset numbered GSE206848, whose data sequencing platform was GPL570, was selected by screening, including 7 cases of normal non-arthritic samples and 7 cases of arthritic samples. The matrix data were transformed into gene name matrix expression data using the gene name transformation tool of Sangerbox 3.0.^[12]^ Differential gene analysis was then performed by selecting the “limma” differential analysis tool to determine differential gene expression between OA and NA. The thresholds for differentially expressed genes were set at 1.5-fold and *P* value < .05, with positive numbers indicating up-regulation of differentially expressed genes (DEGs) and negative numbers indicating down-regulation of DEGs. Volcano and heat maps were utilized to show the results of differentially expressed genes.

### 2.2. Weighted gene co-expression network analysis

The OA gene co-expression network of GSE206848 was constructed using the weighted gene co-expression network analysis package of Sangerbox 3.0. Based on the gene expression profiles, the top 50% of genes with the smallest median absolute deviation were calculated and excluded. Then the correlation coefficients between each gene pair were calculated to construct a similarity matrix, and a suitable soft threshold was chosen to convert the similarity matrix into a neighbor-joining matrix to construct a scale-free network. Subsequently, a topological overlap matrix was created to measure the average network connectivity of each gene. Based on the weighted correlation, a hierarchical cluster analysis was performed and the genes with similar expression profiles were grouped into different modules using a dynamic tree-cutting method based on the set criteria to cut the clustering results, which were represented by the branches of the clustering tree and different colors. The first principal component of the gene expression profile of each module is called the module characteristic gene (ME), which is used to assess the association between modules and phenotypes, where the module with the highest absolute value of the correlation coefficient is the key module that needs to be further analyzed. Module membership (MM) is the correlation coefficient between the expression value of the gene and the ME of the module, representing the correlation between the gene and the module. Gene significance (GS) is the correlation coefficient between the expression value of a gene and the phenotype, representing the correlation between the gene and the phenotype. The MM threshold was set to 0.8, GS threshold to 0.1, weight threshold to 0.1, and all module Hub genes were extracted.^[13]^

### 2.3. OA disease gene acquisition

The GeneCards (https://www.genecards.org/) illness database was searched using the term “osteoarthritis” to find the target proteins of the condition to investigate the targets and functions of the condition.^[14]^ Following that, GeneCards, 1.5-fold differential genes, and Hub hubs were intersected. At first, the intersected genes were identified as crucial hub genes for the onset of OA. Protein–protein interactions (PPI) are critical for understanding cellular physiology in both normal and disease states, so PPI network analysis was performed on the obtained set of intersecting genes using the string database (http://string-db.org/). Species selection was set to “Homo sapiens” with a confidence value > 0.4. PPI networks were constructed by Cytoscape software (version 3.10.1).^[15]^ In addition, the CytoHubba algorithm in Cytoscape software was used to construct the OA gene regulatory network to further screen the top 10 key genes.

### 2.4. Validation of key genes and pathways

Based on the dataset, we identified the critical gene expression findings for statistical analysis to confirm the validity of the genes linked to OA. To further clarify the major signaling pathways involved in the regulation of these genes, possible hub target genes were further submitted to GSEA (Gene set enrichment analysis) study.^[16]^ We obtained the GSEA software (version 3.0) from the GSEA website (http://software.broadinstitute.org/gsea/index.jsp), divided the samples into high-expression group (≥50%) and low-expression group (<50%) based on the expression levels of the extracted hub genes, respectively, and downloaded the c2.cp.kegg.v7.4.symbols.gmt subset from Molecular Signatures Database (http://www.gsea-msigdb.org/gsea/downloads.jsp) for assessing related pathways and molecular mechanisms, setting a minimum gene set of 5 and a maximum gene set of 5000, 1000 resampling, *P* value < .05, and FDR < 0.25 were considered statistically significant.

### 2.5. Enrichment analysis of OA crosstalk genes

Gene enrichment analysis was performed on the intersecting genes of OA to examine the pertinent signaling pathways and processes of the condition.^[17]^ The enrichment analysis was chosen using the species restriction “H.sapiens” after the gene set was imported into the OECloud platform (https://cloud.oebiotech.com). Once the crucial gene set’s Gene Symbol was input and submitted in the common parameters, the results were shown in various tables or charts.

### 2.6. Identification of immune infiltrating cells in OA

Immune cell infiltration is a regulatory mechanism of the immune system during disease development, which generally consists of immune cells, inflammatory cells, fibroblasts, cytokines, and chemokines, and this analysis is an important guideline for predicting the course of the disease and response to treatment. We calculated the immune infiltrating cell score for each sample using the quanTIseq, MCPCounter, xCell, and EPIC algorithms in Sangerbox 3.0, respectively. quanTIseq is a method to quantify the fraction of 10 immune cell types from large amounts of RNA sequencing data^[18]^; MCPCounter allows for robust quantification of the absolute abundance of 8 immune cell populations and 2 stromal cell populations in heterogeneous tissues from transcriptome data^[19]^; xCell is a novel method based on gene characterization and its use to infer 64 immune cell and stromal cell types^[20]^; EPIC based on the reformulation of cell type-specific mRNA content and the ability to consider uncharacterized and potentially highly variable cell types.^[21]^ Finally, difference box line plots were used to show statistical differences between different immune cells.

### 2.7. Hub gene-based drug prediction

To anticipate associated therapeutic medication ingredients, we used the BioCloud platform’s key gene set predictive medication analysis tool based on the 10 Hub genes. Using searches in the drug database DrugBank (5.1.12) and the traditional Chinese medicine database, we identified the main active compounds within the effective distance and examined their mechanisms of action.

## 3. Results

### 3.1. Results of differential gene limma analysis based on different subgroups

**Figure 1.**
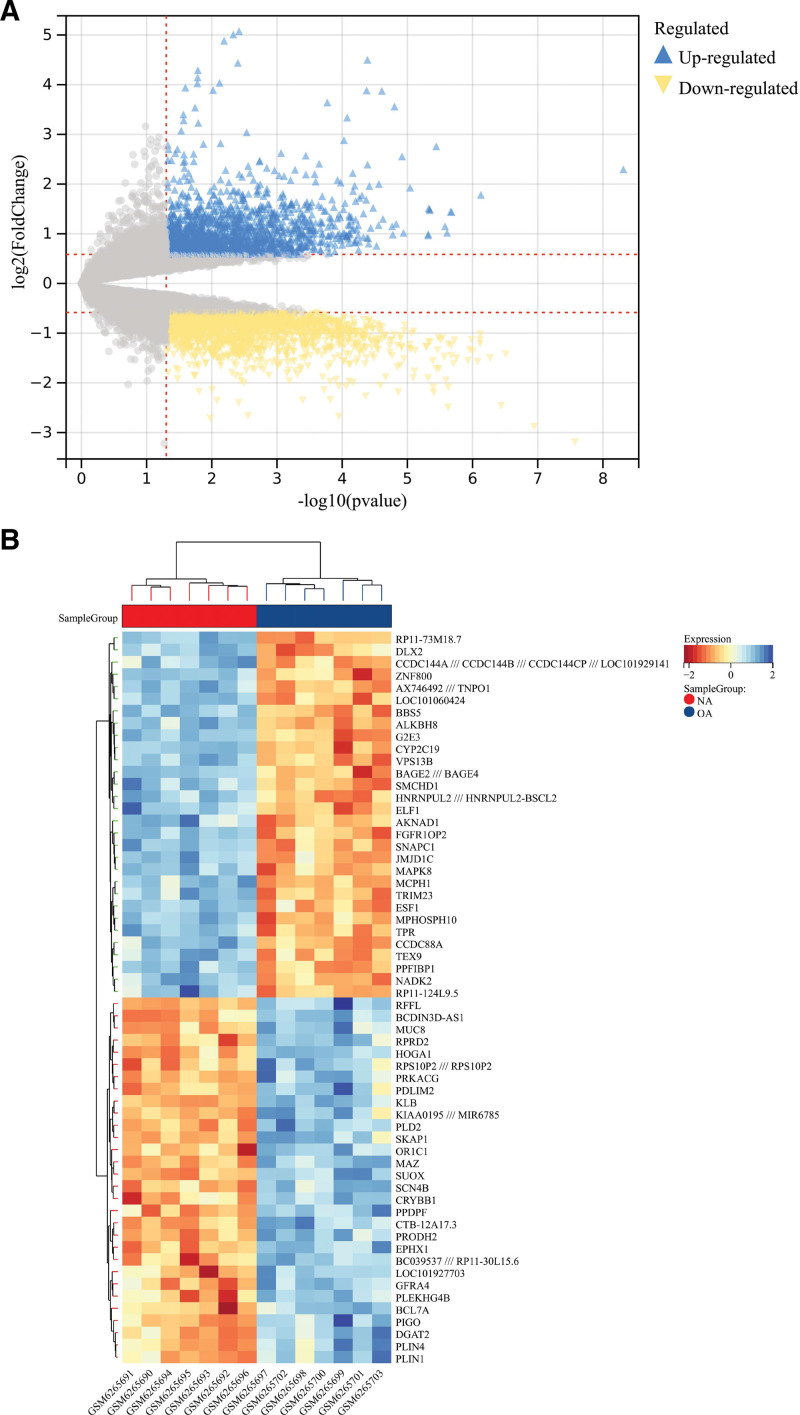
Results of differential gene limma analysis based on different subgroups. NA = no-osteoarthritis, OA = osteoarthritis.

Seven NA and 7 OA samples were selected from the dataset GSE206848 for this analysis. The database download data were transformed and organized to obtain a standard expression matrix dataset with gene names (Table S1, Supplemental Digital Content, http://links.lww.com/MD/M763). Differential research on OA samples and normal control samples revealed 3756 DEGs out of 23,518 genes, of which 1679 were up-regulated and 2077 were down-regulated (Fig. 1A, Table S2, Supplemental Digital Content, http://links.lww.com/MD/M764), and Figure 1B shows the results of the top 30 heatmaps for the differential genes.

### 3.2. Weighted gene co-expression network analysis results

**Figure 2.**
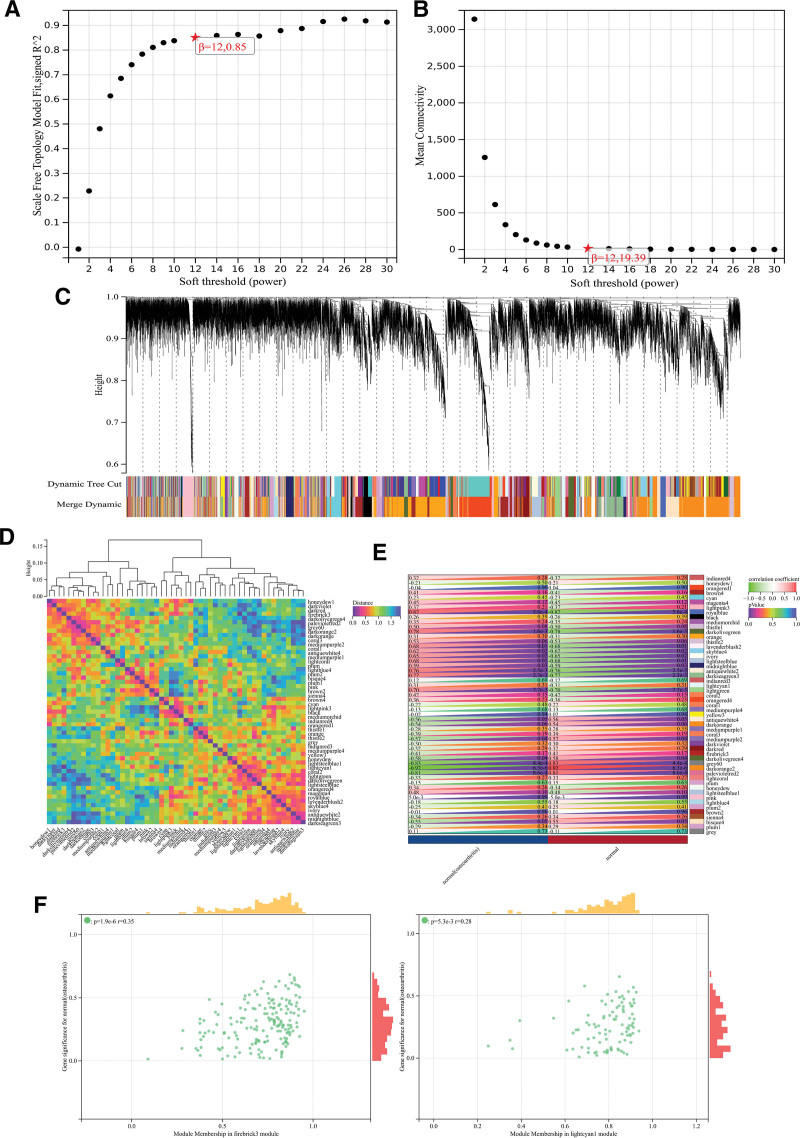
Results of weighted gene co-expression network analysis.

After the gene expression profiles were organized, the expression profiles of 23,518 genes were extracted from the gene matrix and used to construct a weighted gene co-expression network analysis. When the soft threshold power was set to 12, the scale independence reached 0.85 and the average connectivity value was 19.39 (Fig. 2A, B). When the module merging threshold was set to 0.25, the minimum module size was set to 30, and the sensitivity was set to 3, 53 different co-expression modules were obtained by dynamic tree cutting (Fig. 2C). Among the individual modules a module feature vector clustering analysis was performed showing firebrick3 with lightcyan1 module Distance was the largest (Fig. 2D). Then, correlation analysis of the modules with clinical characteristics revealed that the firebrick3 module was negatively correlated with OA (correlation coefficient = −0.41, *P* value = .17) and the lightcyan1 module was positively correlated with OA (correlation coefficient = 0.31, *P* value = .31; Fig. 2E). In addition, correlation analysis between MM and GS showed that both module genes and phenotypes were highly correlated (*r* = 0.35, *P* = 1.9e-6; *r* = 0.28, *P* = 5.3e-3, Fig. 2F). Finally, extracting all the modular Hub genes, we obtained a total of 3505 modular genes (Table S3, Supplemental Digital Content, http://links.lww.com/MD/M765).

### 3.3. OA gene interaction results

**Figure 3.**
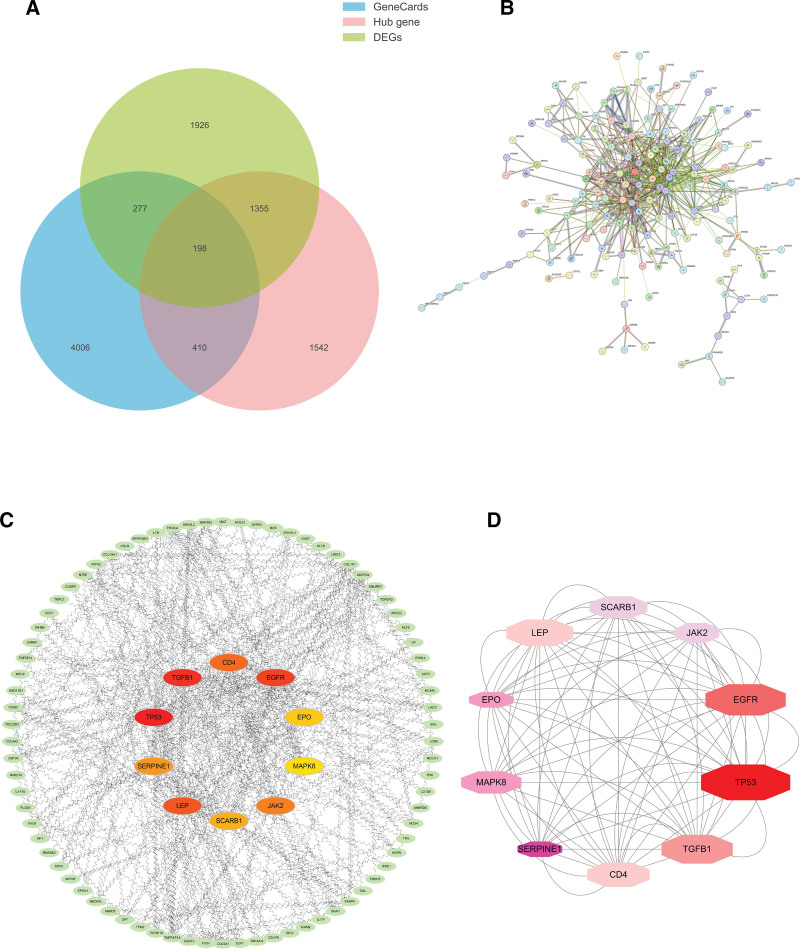
Osteoarthritis gene interaction results and protein–protein interactions results. DEGs = differentially expressed genes.

Firstly, we searched in GeneCards disease database and obtained 4891 target proteins for OA action (Table S4, Supplemental Digital Content, http://links.lww.com/MD/M766). GeneCards, differential gene set, and modular Hub gene set took the intersection to obtain 198 key genes for OA action (Table S5, Supplemental Digital Content, http://links.lww.com/MD/M767, Fig. 3A). The interprotein interaction analysis network showed the correlation between these 198 genes (Fig. 3B), and after further screening by algorithmic scoring we obtained the top 10 OA-associated hub genes (Fig. 3C). These 10 hub genes and their interactions included TP53, EGFR, TGFB1, LEP, CD4, MAPK8, SCARB1, ADIPOQ, JAK2, and SERPINE1 (Fig. 3D).

### 3.4. Dataset-based target validation

**Figure 4.**
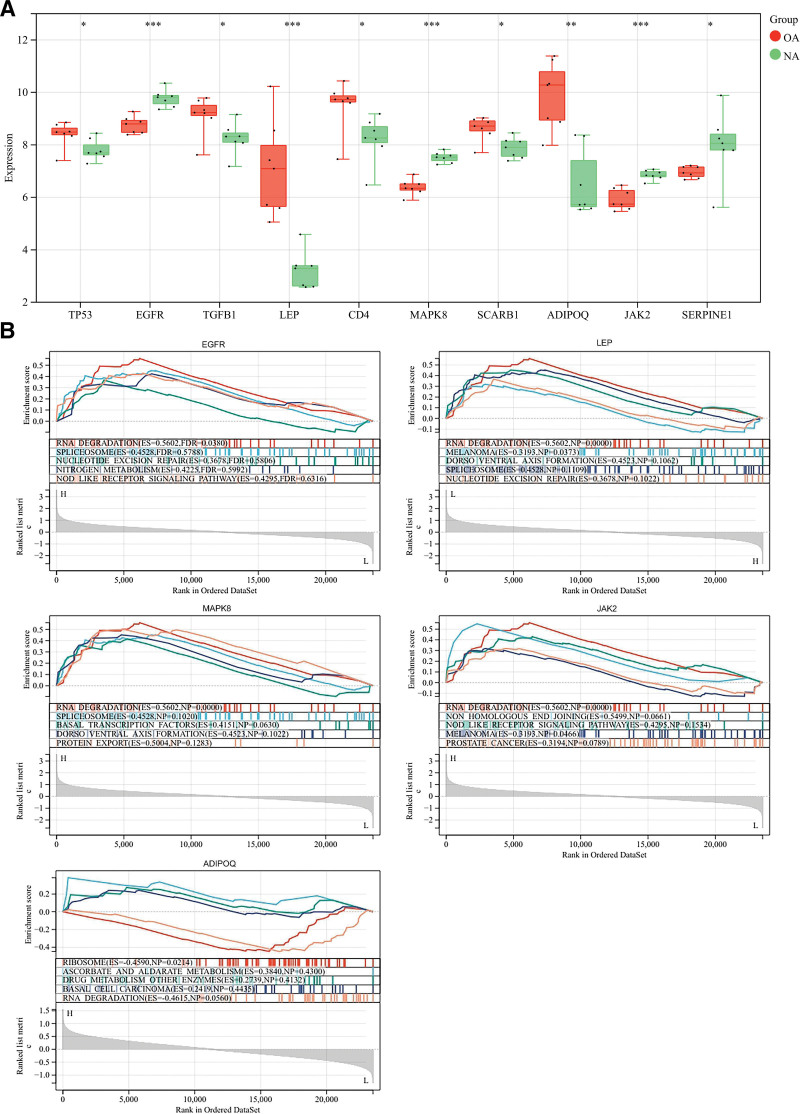
Dataset-based validation of key targets. NA = no-osteoarthritis, OA = osteoarthritis.

Initial research revealed that key genes regulating cellular amino acid metabolism, inflammatory immune response, and OA development include TP53, EGFR, TGFB1, LEP, CD4, MAPK8, SCARB1, ADIPOQ, JAK2, and SERPINE1. We validated using the dataset to see if the relevant hub genes are believable. Each of these hub genes was shown to be statistically significant (*P < .05) according to statistical analysis; hub genes with higher statistical significance (**P < .01) included LEP, MAPK8, EGFR, ADIPOQ, and JAK2 (Fig. 4A). In addition, we performed GSEA analysis using the dataset based on statistically significant pivotal genes, which showed that LEP, MAPK8, EGFR, ADIPOQ, and JAK2 were enriched in metabolic, inflammatory, immune, and senescence signaling pathways (Fig. 4B, Table S6, Supplemental Digital Content, http://links.lww.com/MD/M768).

### 3.5. Intersecting gene enrichment analysis results

**Figure 5.**
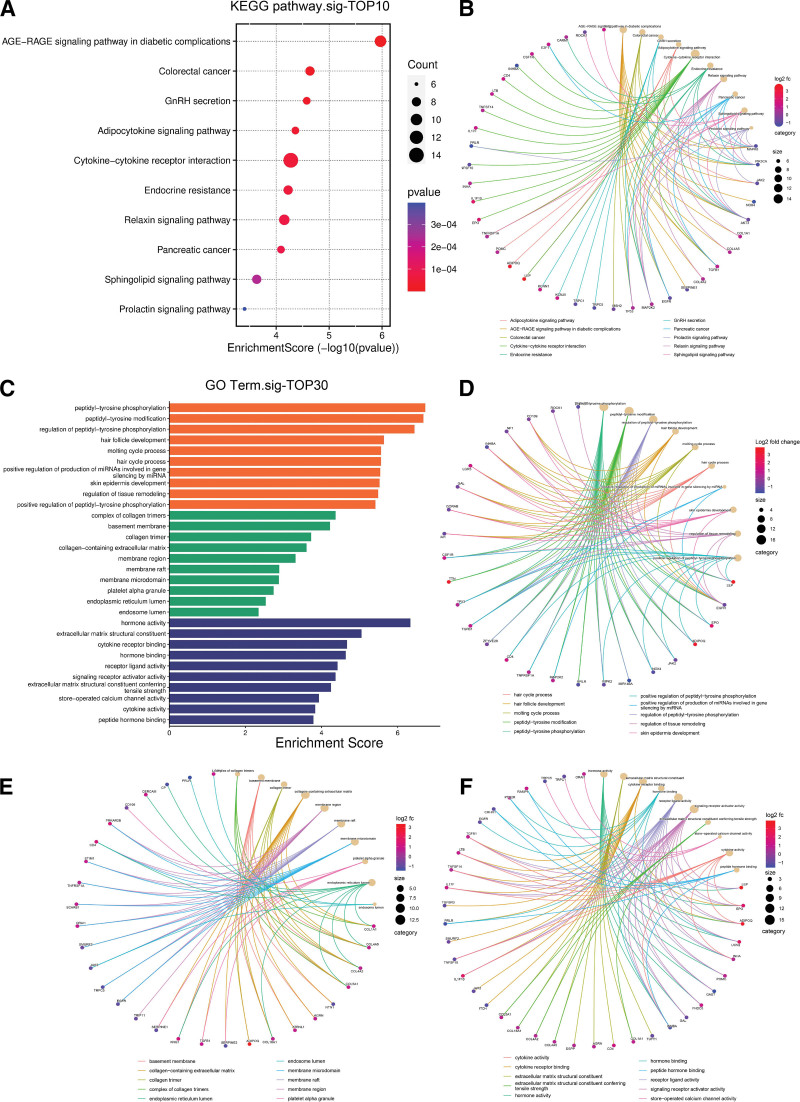
Results of KEGG and GO enrichment analysis of intersecting genes.

Based on the enrichment analysis of 198 OA key intersecting genes, KEGG signaling pathway was enriched to 236, which mainly involved Adipocytokine signaling pathway, Cytokine-cytokine receptor interaction, Endocrine resistance, Relaxin signaling pathway, EGFR tyrosine kinase inhibitor resistance (Fig. 5A, B, Table S7, Supplemental Digital Content, http://links.lww.com/MD/M769). The GO enrichment results showed a total of 4439 entries, which were mainly categorized into Biological Process, Cellular Component, Molecular Function (Table S8, Supplemental Digital Content, http://links.lww.com/MD/M770). Biological process is primarily concerned with regulation of peptidyl-tyrosine phosphorylation, regulation of tissue remodeling, regulation of endocrine process, macrophage differentiation, positive regulation of reactive oxygen species metabolic process, cellular response to peptide. Cellular component consists mainly of complex of collagen trimers, collagen-containing extracellular matrix, membrane region, endoplasmic reticulum lumen, banded collagen fibril, transmembrane transporter complex, striated muscle thin filament. Molecular function is mostly concerned with hormone activity, extracellular matrix structural constituent, cytokine receptor binding, receptor ligand activity, signaling receptor activator activity, cytokine activity, protease binding, endopeptidase regulator activity, peptide hormone receptor binding (Fig. 5C–F). Based on the complete enrichment findings, we ultimately concluded that, in addition to inflammation, immunological control also plays a significant role in the development of OA and that it is strongly connected to amino acid metabolism, endocrine metabolism, cellular senescence, etc.

### 3.6. Analysis of immune cell infiltration results

**Figure 6.**
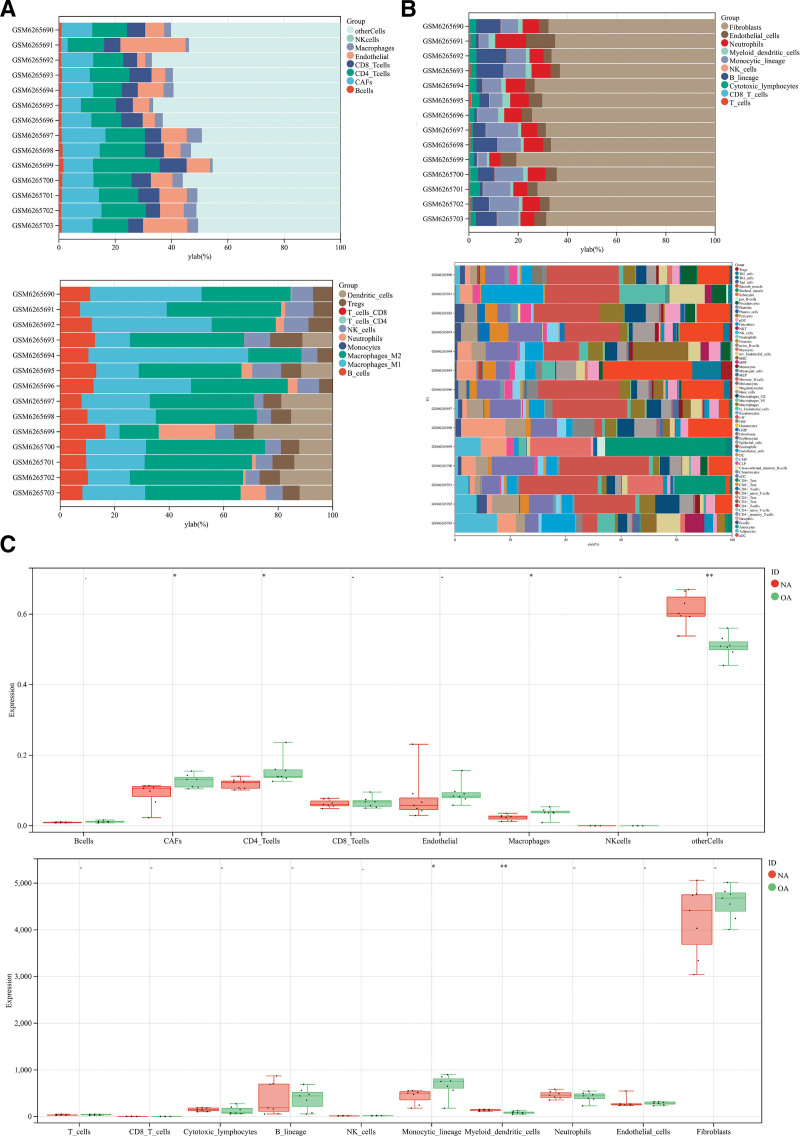
Results of OA immune cell infiltration analysis. NA = no-osteoarthritis, OA = osteoarthritis.

**Figure 7.**
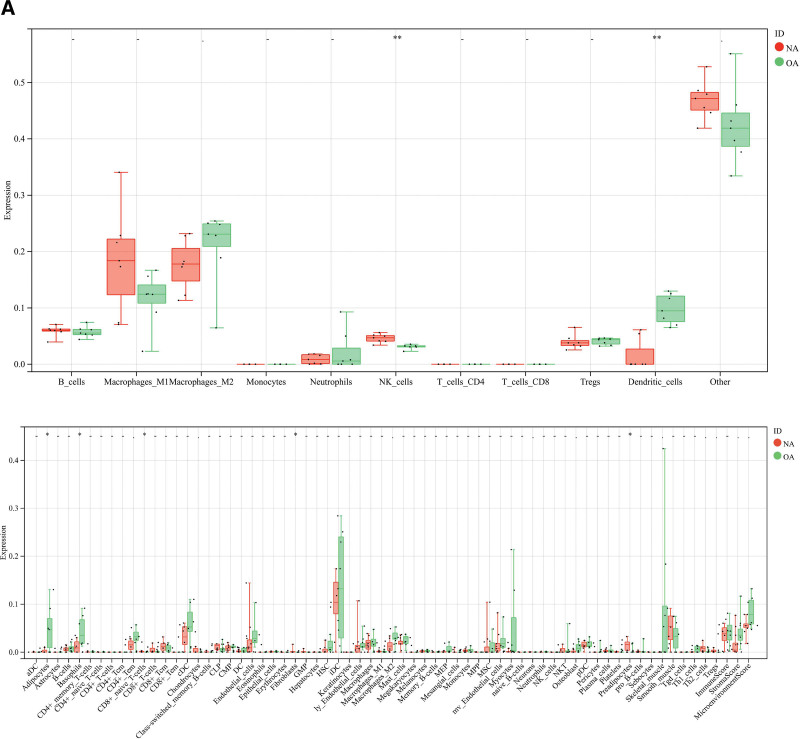
Results of OA immune cell infiltration analysis. NA = no-osteoarthritis, OA = osteoarthritis.

We obtained the immune infiltration scores of different potential therapeutic genes by quanTIseq, MCPCounter, xCell, and EPIC algorithms, respectively, and the results of the immune infiltration scores were demonstrated with stacked plots (Table S9, Supplemental Digital Content, http://links.lww.com/MD/M771, Fig. 6A). The immune infiltration stacked plot showed that the main immune cells involved in OA genes were NK_cells, CD4_Tcells, Macrophages, Monocytic_lineage, Dendritic_cells, Basophils, CD8+_naive_T-cells, Myeloid_dendritic_cells (Figs. 6B and 7A). Figures 6B and 7A show the correlation of different immune cells and the statistical significance among different subgroups. The results showed statistically significant differences among NK_cells, Dendritic_cells, and Myeloid_dendritic_cells (**P < .01).

### 3.7. Potential key drug projections

**Figure 8.**
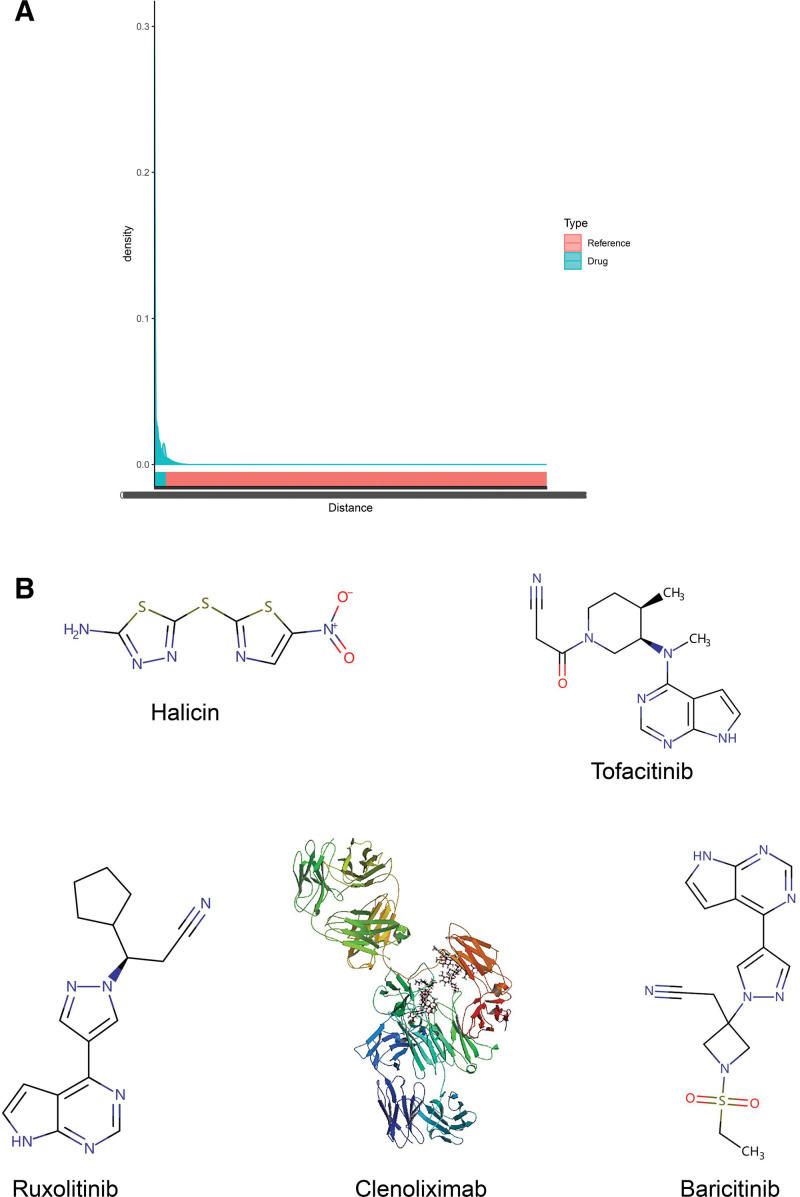
Potential key drug predictions and drug molecular structures for the treatment of osteoarthritis.

To explore potential drugs targeting key gene therapy for OA, we first predicted 76 relevant compounds in the Key Gene Set Predictive Drug Analysis tool based on 10 pivotal genes (Fig. 8A, Table S10, Supplemental Digital Content, http://links.lww.com/MD/M772). Among them, Distance ranking combined with literature search, the main 5 compounds we screened were Halicin, Ruxolitinib, Tofacitinib, Clenoliximab, and Baricitinib (Fig. 8B).

## 4. Discussion

An orthopedic condition called OA is brought on by a number of variables, including hereditary, environmental, and metabolic ones. The molecular causes of OA have first been disclosed by research on biological processes such as inflammation response, abnormalities of cartilage metabolism, and joint degradation.^[22]^ The diagnosis and assessment of early OA is limited by the limits of traditional approaches, which include clinical symptoms, physical examination, and imaging manifestations.^[23]^ Screening and validation of biomarkers are intended to give a better reference for early intervention and tailored therapy, hence improving the sensitivity and accuracy of diagnosis. Furthermore, non-pharmacological therapies like physical activity and physiotherapy as well as pharmaceutical therapies like non-steroidal anti-inflammatory medicines and intra-articular injections are the key components of conventional treatments.^[24]^ These techniques, however, merely address symptoms and have little impact on OA disease progression and structural restoration. Thus, it is also very important to develop novel therapeutic modalities, such as stem cell therapy and biologics.

Using the GEO database, we were able to identify 3756 genes that were differently expressed in OA patients compared to normal samples by gene expression analysis. In OA, 198 relevant intersecting genes that are differently expressed have been found. TP53, EGFR, TGFB1, LEP, CD4, MAPK8, SCARB1, ADIPOQ, JAK2, and SERPINE1 were shown to be significant hub genes by further screening. Enrichment analysis revealed that immunological response, aging, and amino acid metabolism, in addition to inflammation, are the key factors contributing to the development of OA.

Cellular senescence is a cyclic form of the cell cycle in which cells remain viable and in a no proliferative state despite exposure to mitogenic signaling stimuli. Research suggests that cellular senescence is closely related to age, which is an important factor in the mechanism of OA.^[25]^ Like other organs, joint tissues senesce and degenerate with age, and the number of chondrocytes and synovial fibroblasts in the joints is closely related to age, that is, aging stimulates cartilage degradation. Conversely OA can also induce phenotypic changes in joint cells associated with senescence characteristics, characterized mainly by chondrocyte degeneration and disintegration of the extracellular matrix. For example, the cell surface protein urokinase fibrinogen activator surface receptor is widely induced in senescent cells, which include chondrocytes derived from those in OA.^[26]^ Most senescent joint cells exhibit common features such as telomere erosion, increased expression of p53 and cell cycle protein-dependent kinase inhibitor p21.^[27]^ In addition, senescence induces cellular metabolic remodeling, and these remodeling may promote OA progression. Research has found that senescent fibroblasts induce cartilage erosion and loss of mobility in the knee joint of mice, suggesting that senescent cells alter the intra-articular microenvironment and induce OA-like arthropathy.^[28]^

The immune system is a defense function formed during the development and evolution of the organism, and it is also involved in the removal of damaged, apoptotic, and senescent cells from the body as well as the initiation and regulation of the immune response process. The conventional immune system is mainly composed of barrier structures, monocytes, macrophages, natural killer cells, T cells, B cells, complement and a series of cytokines.^[29]^ Several researches have shown that the activation and action of infiltrating immune cells play an important role in the formation of inflammation and sustained cartilage tissue damage in OA synovial tissue.^[30]^ However, the specific mechanism of the immune system involved in the development of OA inflammation is still unclear. Therefore, we further researched the relationship between OA and immune cell infiltration, i.e., we obtained comprehensive results of immune infiltration by quanTIseq, MCPCounter, xCell, and EPIC algorithms, respectively. Further investigation revealed that immune cell infiltration was increased in the OA group compared with the normal group, such as NK_cells and Dendritic_cells. For example, research has found that high levels of NK cells are associated with the clearance of inflammatory factors, suggesting that patients have progressed to the stage of the inflammatory cycle, i.e., they are significantly characterized by elevated levels of activated NK cells in the OA group compared to the normal group.^[31]^ NK cells in the synovium express granzyme B and perforin to damage synovial cell, while the environmental NK motif 2D ligand Rae-1 is activated, making it a target for NK cells. In addition, NK motif 2D promotes the production of pro-inflammatory effects of Th1 and Th17, causing antigen-induced Guillain-Barré.^[10]^ The infiltration of these immune cells is an important cause of various inflammatory conditions, so treating OA in terms of immune modulation may also be a new therapeutic target. We will further search for suitable drugs to modulate immune-related targets for the treatment of OA in subsequent research.

Based on the key drug prediction of hub genes, we initially analyzed 76 potential compounds and further screened Halicin, Ruxolitinib, Tofacitinib, Clenoliximab, Baricitinib and several others that may serve as potential drugs for the treatment of OA. Tofacitinib, Clenoliximab is an effective drug that has been shown to be used in the treatment of arthritis. Research has found that Tofacitinib is able to keep the expression of inflammatory factors to a certain extent, thus reducing the damage of inflammation to articular chondrocytes.^[32]^ Clenoliximab is a chimeric monoclonal antibody against CD4 from rhesus monkeys, which has immunomodulatory effects.^[33]^ Baricitinib is a Janus kinase (JAK) inhibitor used for the treatment of moderate to severe rheumatoid arthritis that does not respond well to at least one TNF antagonist. JAK is a tyrosine protein kinase that plays an important role in pro-inflammatory signaling pathways.^[34]^ Overactive JAK is associated with autoimmune diseases such as rheumatoid arthritis. Baricitinib attenuates JAK-mediated inflammatory and immune responses by inhibiting the actions of JAK1 and JAK2.^[35]^ Therefore, the screening of these 3 drugs has some scientific significance. The current state of research on Ruxolitinib and Halicin in arthritis is unclear. An antibiotic called Halicin demonstrates strong bactericidal action against a wide range of pathogens across several evolutionary branches, such as carbapenem-resistant Enterobacteriaceae and Mycobacterium tuberculosis.^[36]^ An effective treatment for a variety of bone marrow fibrosis types and steroid therapy-refractory graft-versus-host disease is Rufolitinib, a kinase inhibitor. Studies have shown that Roxolitinib, by decreasing JAK expression, can somewhat relieve chronic low-grade inflammation.^[37]^ Therefore, both may serve as potential drugs for the treatment of OA, which may be a research direction we need to explore further. Based on the above drug prediction and related literature review, it is evident that drug prediction based on hub genes has some scientific significance and is of guiding significance for clinical as well as basic research.

## 5. Conclusion

In summary, based on the pivotal genes, we established an OA disease prediction model, and found that the pathogenesis of OA patients is mainly related to the aberrant expression of cellular senescence, inflammation, metabolism, immune regulation and other signaling pathways. Finally, we predicted that Halicin, Ruxolitinib, Tofacitinib, Clenoliximab, and Baricitinib may be the key drugs or components for the treatment of OA. However, our research has some limitations; it still lacks relevant information about the clinical characteristics of the patients, such as age, gender, and information about the underlying disease, which may affect gene expression. Furthermore, future research should include in vitro and in vivo experiments to validate the therapeutic potential of the identified compounds.

## Author contributions

**Conceptualization:** Li Zhong.

**Data curation:** Yan Liu, Li Zhong.

**Methodology:** Yan Liu, Juan Huang.

**Software:** Wei Jiang.

**Visualization:** Juan Huang.

**Writing – original draft:** Yan Liu.

**Writing – review & editing:** Wei Jiang, Li Zhong.

## Supplementary Material




















